# Baseline reef health surveys at Bangka Island (North Sulawesi, Indonesia) reveal new threats

**DOI:** 10.7717/peerj.2614

**Published:** 2016-10-25

**Authors:** Massimo Ponti, Francesca Fratangeli, Nicolò Dondi, Marco Segre Reinach, Clara Serra, Michael J. Sweet

**Affiliations:** 1 Dipartimento di Scienze Biologiche, Geologiche e Ambientali, University of Bologna, Ravenna, Italy; 2 Reef Check Italia onlus, Ancona, Italy; 3 Coral Eye, Bangka Island, North Sulawesi, Indonesia; 4 Environmental Sustainability Research Centre, University of Derby, Derby, United Kingdom

**Keywords:** Coral diseases, Coral bleaching, Indo-Pacific, Scleractinians, Terpios hoshinota, Chalinula nematifera, Waminoa sp., Cryptochirid crabs, Pyrgomatid barnacles, Skeletal eroding band

## Abstract

Worldwide coral reef decline appears to be accompanied by an increase in the spread of hard coral diseases. However, whether this is the result of increased direct and indirect human disturbances and/or an increase in natural stresses remains poorly understood. The provision of baseline surveys for monitoring coral health status lays the foundations to assess the effects of any such anthropogenic and/or natural effects on reefs. Therefore, the objectives of this present study were to provide a coral health baseline in a poorly studied area, and to investigate possible correlations between coral health and the level of anthropogenic and natural disturbances. During the survey period, we recorded 20 different types of coral diseases and other compromised health statuses. The most abundant were cases of coral bleaching, followed by skeletal deformations caused by pyrgomatid barnacles, damage caused by fish bites, general pigmentation response and galls caused by cryptochirid crabs. Instances of colonies affected by skeletal eroding bands, and sedimentation damage increased in correlation to the level of bio-chemical disturbance and/or proximity to villages. Moreover, galls caused by cryptochirid crabs appeared more abundant at sites affected by blast fishing and close to a newly opened metal mine. Interestingly, in the investigated area the percentage of corals showing signs of ‘common’ diseases such as black band disease, brown band disease, white syndrome and skeletal eroding band disease were relatively low. Nevertheless, the relatively high occurrence of less common signs of compromised coral-related reef health, including the aggressive overgrowth by sponges, deserves further investigation. Although diseases appear relatively low at the current time, this area may be at the tipping point and an increase in activities such as mining may irredeemably compromise reef health.

## Introduction

Coral reefs around the world are increasingly threatened by a multitude of stressors, both natural and anthropogenic. These include decline in water quality, overexploitation of resources and global climate change which have all been linked with the onset of mass coral bleaching and a variety of different disease signs (Carpenter et al., 2008; Ban, Graham & Connolly, 2014; Burge et al., 2014; Thompson et al., 2014). Many environmental stressors and anthropogenic disturbances are thought to favour the onset of infectious disease, either on their own or more commonly synergistically (Sutherland, Porter & Torres, 2004; Harvell et al., 2007). For example, anomalous high sea surface temperatures and their increasing frequency have been shown to raise coral susceptibility and pathogen virulence, influencing the severity and rate of spread of infections (Harvell et al., 2002); (Lafferty & Holt, 2003; Randall et al., 2014; Wooldridge, 2014). Furthermore, disease susceptibility has also been linked to high sedimentation rates, water turbidity and eutrophication (Bruckner & Bruckner, 1997; Bruno et al., 2003; Fabricius, 2005; Voss & Richardson, 2006a; Voss & Richardson, 2006b; Haapkylä et al., 2011; Pollock et al., 2014). Interestingly, in controlled experiments the above stressors have proven insufficient to cause the onset of disease without direct physical injury. Such injury has been shown to occur from contact with macroalgae, direct physical damage and predation for example (Nugues et al., 2004; Nicolet et al., 2013; Séré et al., 2015). For this reason, it has been strongly recommended that any coral reef health monitoring undertaken should consider all possible sign of compromised health and not only those of infectious diseases and bleaching (Raymundo, Couch & Harvell, 2008).

Baseline coral health surveys are an important first step in identifying areas of concern where management and mitigation strategies need to be implemented. To date, the majority of published studies on coral diseases have been focused around the Caribbean (for a review see Weil & Rogers, 2011), and the Australian Great Barrier Reef (e.g., Willis, Page & Dinsdale, 2004; Haapkylä et al., 2013). More recently, survey effort has increased to cover other areas of the Indo-Pacific such as the Maldives (e.g., Montano et al., 2012; Montano et al., 2013; Montano et al., 2015; Montano et al., 2016) and certain areas within the ‘Coral Triangle’ (e.g., Brown & Suharsono, 1990; Hoeksema, 1991; Haapkylä et al., 2007; Haapkylä et al., 2009b; Nugues & Bak, 2009; Burke et al., 2012; Cervino et al., 2012; Sabdono et al., 2014; Haapkylae, Melbourne-Thomas & Flavell, 2015; Johan, Ginanjar & Priyadi, 2015; Miller et al., 2015). Nevertheless, baseline coral health surveys remain sparse in other locations such as Sulawesi, Indonesia, for example (De Vantier & Turak, 2004; Fava et al., 2009). In this study, we therefore aimed to assess the reef health around Bangka Island, within a small archipelago at the northern tip of Sulawesi.

**Figure 1:**
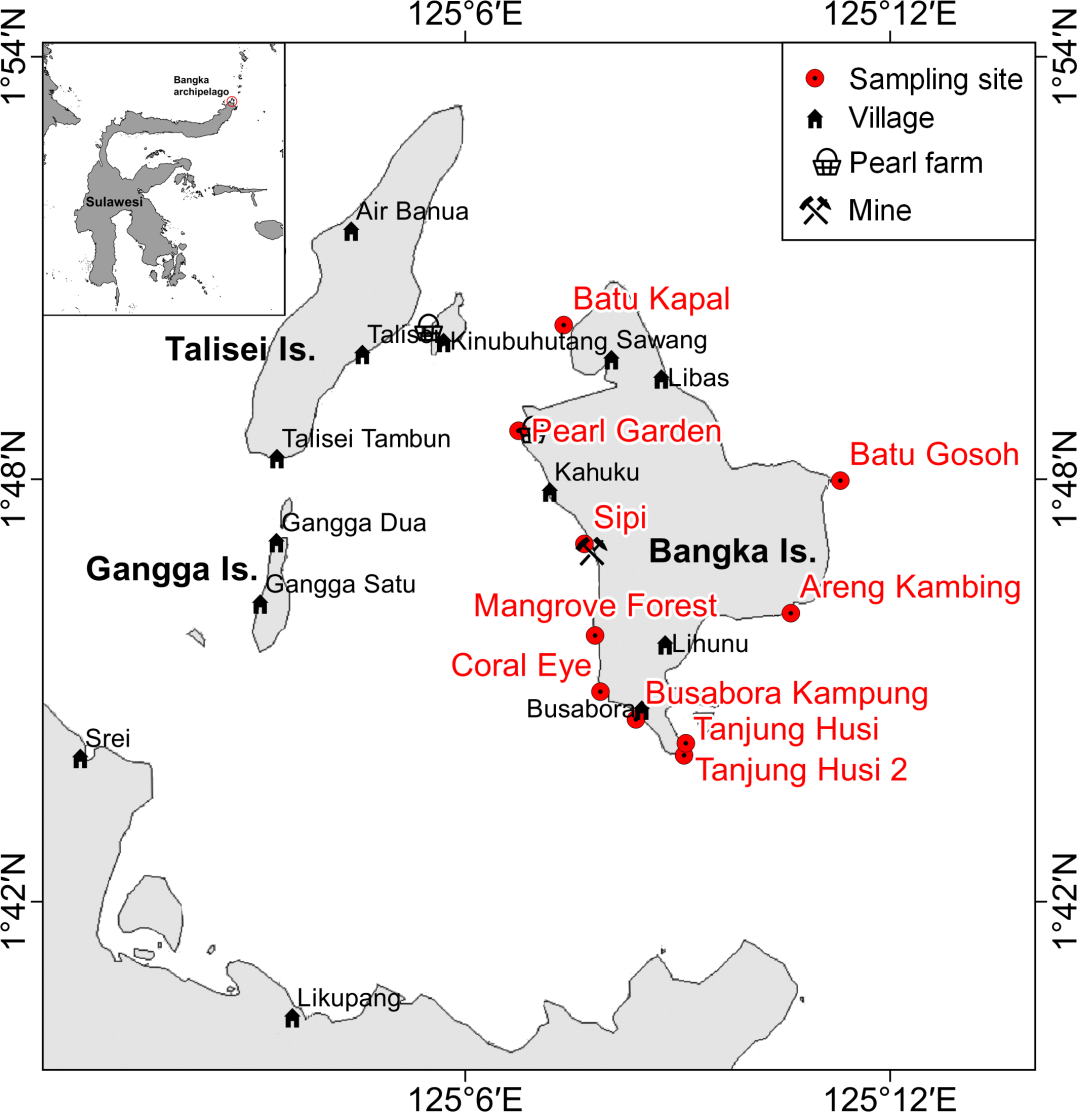
Sketch map of Bangka archipelago (north Sulawesi). Study sites, villages, pearl farms and mine are shown.

**Table 1:** Main characteristics of the study sites. Table 1 reports habitat typologies, disturbance levels, nearest village (including distance and inhabitants), island side, wave exposure, previous storm. Human disturbances were divided into three main groups: m, mechanic (e.g., anchoring, boat strike, SCUBA diving, blastfishing), bc, bio-chemical (e.g., boat engine leaching, villages sewages), f, fishing pressure. Intensities of human disturbances and wave exposure were classified into four ranked levels (0, none; 1, low; 2, medium; 3, high).

Study site	Geographic coordinate WGS84	Habitat	Disturbance			Nearest village (distance- inhabitants)	Island side	Wave exposure	Previous storm
			m	bc	f				
Coral Eye	1.75112°N 125.13334°E	fringing reef	2	0	2	Busabora (1100 m–300)	SW	2	12/11/2012
Busabora Kampung	1.74438°N 125.1401°E	fringing reef	3	1	3	Busabora (100 m–300)	SW	2	12/11/2012
Tanjung Husi	1.73465°N 125.1515°E	volcanic rockslide	1	0	2	Busabora (1800 m–300)	SE	2	unknown
Tanjung Husi 2	1.73752°N 125.15192°E	volcanic rockslide	2	0	2	Busabora (2200 m–300)	SE	2	unknown
Mangrove Forest	1.76303°N 125.13055°E	fringing reef	2	0	3	Kahuku (3600 m–700)	W	2	12/11/2012
Pearl Garden	1.81148°N 125.11253°E	fringing reef	3	1	3	Kahuku (2000 m–700)	W	1	unknown
Batu Gosoh	1.79968°N 125.18828°E	volcanic cliff	1	0	2	Libas (5200 m–500)	E	2	unknown
Sipi	1.78582°N 125.13025°E	fringing reef	3	0	3	Kahuku (1500 m–700)	W	2	12/11/2012
Batu Kapal	1.8365°N 125.12317°E	fringing reef	2	0	3	Kahuku (5000 m–700)	NW	1	unknown
Areng Kambing	1.76882°N 125.17628°E	volcanic cliff	1	0	2	Lihunu (3300 m–1000)	SE	1	unknown

## Materials and Methods

### Study area

Bangka Island belongs to a small archipelago located at the northern tip of Sulawesi, Indonesia (Fig. 1). These islands are exposed to the main current coming from the western Pacific Ocean and directed toward the Indian Ocean (Tomascik et al., 1997). The central area of the archipelago is shallow, while the outer sides drop rapidly to over 1,000 m depth. The islands are covered by lush vegetation and fringing reefs are alternated with mangroves and volcanic cliffs. The islands are home to some villages and few resorts, of which the oldest was built in 1987. Bangka, the largest of these islands is less than 48 km^2^. It has a resident population of about 2,500 inhabitants (as of 2013), distributed throughout five main villages (Busabora, Libas, Kahuku and Lihunu, Sawang), four small resorts in the southeastern side (Murex, Blue Bay, Nomad, Mimpi Indah), and a private research station (the Coral Eye) which hosts researchers and occasional tourists. Threats faced by the reefs in this area include destructive fishing activities (e.g., blast fishing and poison fishing) (De Vantier & Turak, 2004) and mining which targets iron ore and other minerals. Such trends are similar to those faced by other reefs throughout Indonesia (e.g., Edinger et al., 2008; Caras & Pasternak, 2009; Lasut et al., 2010; Edinger, 2012; Reichelt-Brushett, 2012). At Bangka Island, the mine (managed by a foreign company) started in 2013 but has since closed in July 2015. The closure, sanctioned by the Supreme Court in Jakarta was a direct response to the opposition of residents and tourism operators.

**Table 2:** Investigated coral diseases and other signs of compromised health. Adopted classification scheme and acronyms (after Beeden et al., 2008; Raymundo, Couch & Harvell, 2008).

Symptom		Disease or compromised health category	Acronym
Tissue loss	Predation or other stress	Unidentified	NI
		Fish bites	FB
		Crown-of-thorns starfish (*Acanthaster planci*)	COTS
		Gastropod corallivory (e.g., *Drupella* sp.)	GC
	Sediment damage		SD
	Algal overgrowth		AlO
	Coloured band disease	Black band disease	BBD
		Skeletal eroding band	SEB
		Brown band	BrB
	No band	Ulcerative white spot	UWS
		White syndrome	WS
		Atramentous necrosis	AtN
Tissue discolouration	White	Bleaching	BL
		Focal bleaching	FBL
		Non-focal bleaching (e.g., patches, stripes)	NFBL
	Not white	Pigmentation response	PR
		Trematodiasis	TR
Galls caused by cryptochirid crabs			GA
Skeletal deformations caused by pyrgomatid barnacles			BA
Compromised health	Aggressive overgrowth (e.g., coral-killing sponges *Terpios hoshinota* and *Chalinula nematifera*)		AgO
	Acoelomorph flatworm infestation (e.g., *Waminoa* sp.)		RW

### Sampling design and survey method

Benthic assemblages, coral diseases and other signs of compromised health around Bangka Island (North Sulawesi, Indonesia; Fig. 1) were investigated at 10 randomly selected sites at three depths: 3, 6 and 9 m (below the mean lower low water). Surveys were conducted between the months of October and November 2013. Tide levels were calculated by WXTide, a free Windows tide prediction software (http://www.wxtide32.com), using the Manado subordinate station (based on Sungai Kutei reference station, Borneo, Indonesia) and locally calibrated using a depth logger (DST centi-TD from Star-Oddi, http://www.star-oddi.com). Six sites were characterised by fringing reefs, two sites by coral rims growing on volcanic cliffs and the other two by sparse coral heads on volcanic rockslides (see Table 1). Along fringing reefs, the three chosen depths roughly correspond to reef flat, reef crest and slope (i.e., front reef), which are standard subzones in coral reef monitoring studies (e.g., Hill & Wilkinson, 2004; Hoeksema, 2012). At each site and depth, five belt transects (10 × 2 m) were laid randomly along reef contours (Beeden et al., 2008). A gap of at least 5 m was left between each transect. Overall, 150 belt transects were analysed, covering a total area of 3,000 m^2^. Scleractinian coral colonies larger than 20 cm in diameter were identified to genus level across all transects. Scleractinian taxonomy was mainly based on Veron (2000), with some exceptions taking into account the recent reclassification of certain coral species. For example, *Acropora* and *Isopora* genera were separated according to Wallace, Done & Muir (2012). Corals previously from the genus *Favia* were reclassified to the genus *Dipsastraea* (Budd et al., 2012). *Phymastrea* was used to indicate all Indo-Pacific species previously indicated as *Montastrea* in Veron (2000) (according to Budd et al. (2012)) and more recently reassigned to *Astrea*, *Paramontastraea* and *Favites* (Huang et al., 2014). Similarly we have used the functional group ’*Fungia*’ to indicate some subgenera recently elevated to genus level (e.g., *Danafungia*, *Verrillofungia* and *Pleuractis*; see Gittenberger, Reijnen & Hoeksema (2011)).

In order to reduce inconsistencies associated with disease identification (Lindop, Hind & Bythell, 2008), each visible sign of coral disease or other compromised health indicators were photographed, identified and assigned to one of the 21 categories defined according to the identification guides by Beeden et al. (2008) and Raymundo, Couch & Harvell (2008). The classification scheme and adopted category acronyms can be seen in Table 2.

Benthic sessile assemblages, bare rock, sand and coral rubble were quantified by using five photographic samples (50 × 50 cm), which were randomly located within each transect. Benthic organisms were assigned to 14 main groups: algae, encrusting-, massive-, erected- and boring- sponges, hydroids, anemones, soft- and hard- corals, gorgonians, giant clams, colonial-, solitary- and social- ascidians. Percent covers were estimated by superimposing a grid of 100 equal-sized cells, using the software PhotoQuad (Trygonis & Sini, 2012). The data of percent cover was averaged across transects. No hard corals were detected at two transects, both of which were at Pearl Garden.

Information on anthropogenic disturbance sources at each site was obtained by interviewing local people, including those working at the resorts, and from data collected in previous surveys carried out applying the Reef Check protocol (Ponti et al., 2012); freely available data at http://data.reefcheck.us/). Local human disturbances were grouped as mechanic (e.g., anchoring, boat strike, SCUBA diving, blast fishing), bio-chemical (e.g., boat engine leaking, village sewage) and fishing pressure. Storms are the primary natural disturbance in the area. Previous recent storms, such intense as to damage at least the shallow-water corals, were analysed in term of direction, occurrence date and wave exposure at each site. Intensities of human disturbances and wave exposure were classified into four ranked levels according to the maximum impacts recorded in the area (De Vantier & Turak, 2004): 0 = almost no human disturbance and/or well sheltered, 1 = low or occasional disturbance and/or sheltered, 2 = medium intensity, 3 = high and frequent disturbance and/or very exposed.

A research permit was granted prior to undertaking the survey work by the authority of Lihunu, Likupang eastern districts, Minahasa northern district government (Permit ID: 211/2019/SPP/IX-2013 issued at Lihunu on the 28th September 2013).

### Data analyses

In the present study the abundances of each category of coral diseases and other compromised health indicators were expressed for each transect and analysed in terms of mean number of affected colonies per square metre of hard corals, based on the extension of hard corals in the transect.

Differences between sites (10 levels, random) and depths (three levels, fixed) were assessed by two-way crossed univariate and multivariate permutational analysis of variance (PERMANOVA, *α* = 0.05; Anderson & Robinson, 2001; Anderson & Ter Braak, 2003).

Univariate tests were performed on Euclidean distances calculated on untransformed data (Anderson & Robinson, 2001). Multivariate tests were based on Bray-Curtis similarity of square root transformed data (Clarke, 1993). Patterns of similarities in diseases and other signs of compromised health among sites and depths were tested on zero-adjusted Bray-Curtis similarity of square root transformed data. An adjustment was applied to solve the indetermination of the Bray-Curtis coefficient, which occurs when it is calculated for pairs of transects without signs of diseases. This was accomplished by adding a dummy variable with a value of 0.22361, that is the square root of the lowest non-zero value attainable (i.e., 0.05 affected colonies m${}_{\mathrm{hard~ corals}}^{-2}$) (Clarke, Somerfield & Chapman, 2006).

Multivariate similarity patterns were displayed by unconstrained ordination plots using the Principal Coordinate Analysis (PCoA, i.e., metric multidimensional scaling; Gower, 1966) based on the centroids of the similarity clouds at each site. Multivariate multiple regressions between similarity patterns and variables were performed by the DistLM procedure (marginal test; McArdle & Anderson, 2001) and significant correlations (*P* < 0.05) were graphically represented by correlation vectors, proportional to the Pearson’s correlation coefficients, superimposed on the PCoA plots. Correlations between abundances of diseases and other signs of compromised health were made compared with human and natural disturbances by the Spearman’s rank correlation coefficient (*ρ*). *ρ* assesses how well the relationship between two variables can be described using a monotonically increasing or decreasing function. Mean values were always reported together with their standard errors (SE). Statistical analysis was performed using PRIMER 6 with PERMANOVA +add-on package (Anderson, Gorley & Clarke, 2008). Spearman rank correlations and their tests were calculated by the computational language R (R Core Team, 2016).

## Results

### Benthic community structure and coral cover

**Figure 2:**
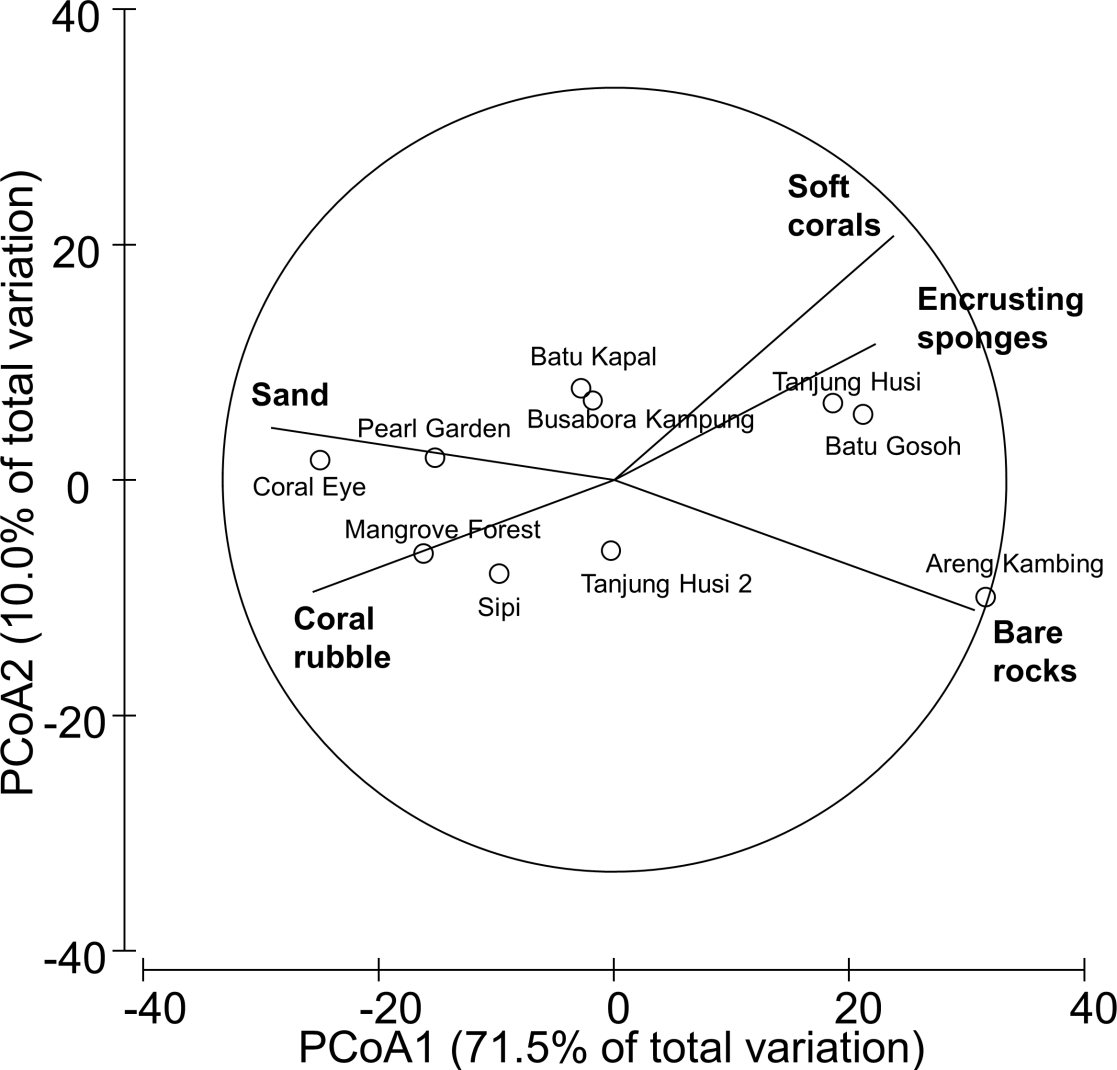
Similarity patterns of benthic assemblages. The PCoA plot shows the multivariate similarity patterns of benthic assemblages, analysed in terms of main groups (i.e., algae, encrusting-, massive-, erected- and boring- sponges, hydroids, anemones, soft- and hard- corals, gorgonians, giant clams, colonial-, solitary- and social- ascidians), among sites. Open circles represent the centroids of the similarities of assemblages found at each site. Superimposed vectors indicate the intensity and direction of the correlations of the benthic variables selected by DistLM procedure (*P* < 0.05 in the marginal test).

**Figure 3:**
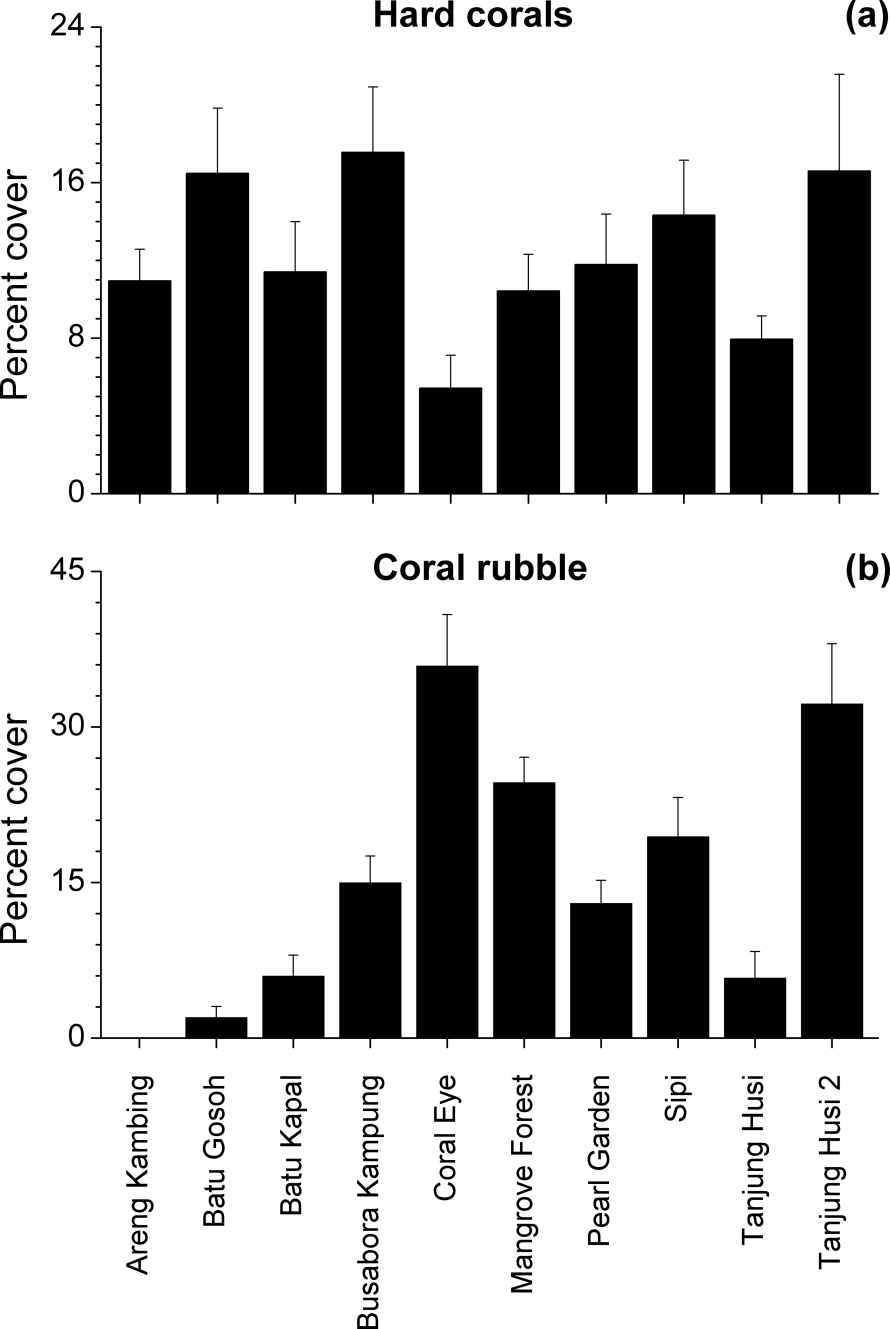
Hard coral and coral rubble abundances. Mean percent cover (+SE) of total hard corals and coral rubble at each study site.

Benthic assemblages were very heterogeneous in the study area and the community structures significantly differed among sites and depths (Site × Depth: *F*_18,120_ = 2.2216, *P* = 0.0001). Differences between sites were significantly related to the percent cover of bare rocks, sand, coral rubble, soft corals and encrusting sponges (Fig. 2). Percent cover of both total hard corals and coral rubble significantly varied among sites but not among depths (Coral rubble, Site: *F*_9,120_ = 16.557, *P* = 0.0001; hard corals, Site: *F*_9,120_= 2.0735, *P* = 0.0340). Total hard coral cover varied between 5.4 ± 1.7% at Coral Eye and 17.6 ± 3.4% at Busabora Kampung (Fig. 3A). Coral rubble cover varied from almost zero at Areng Kambing, a volcanic cliff, to 35.9 ± 5.0% at Coral Eye (Fig. 3B).

**Figure 4:**
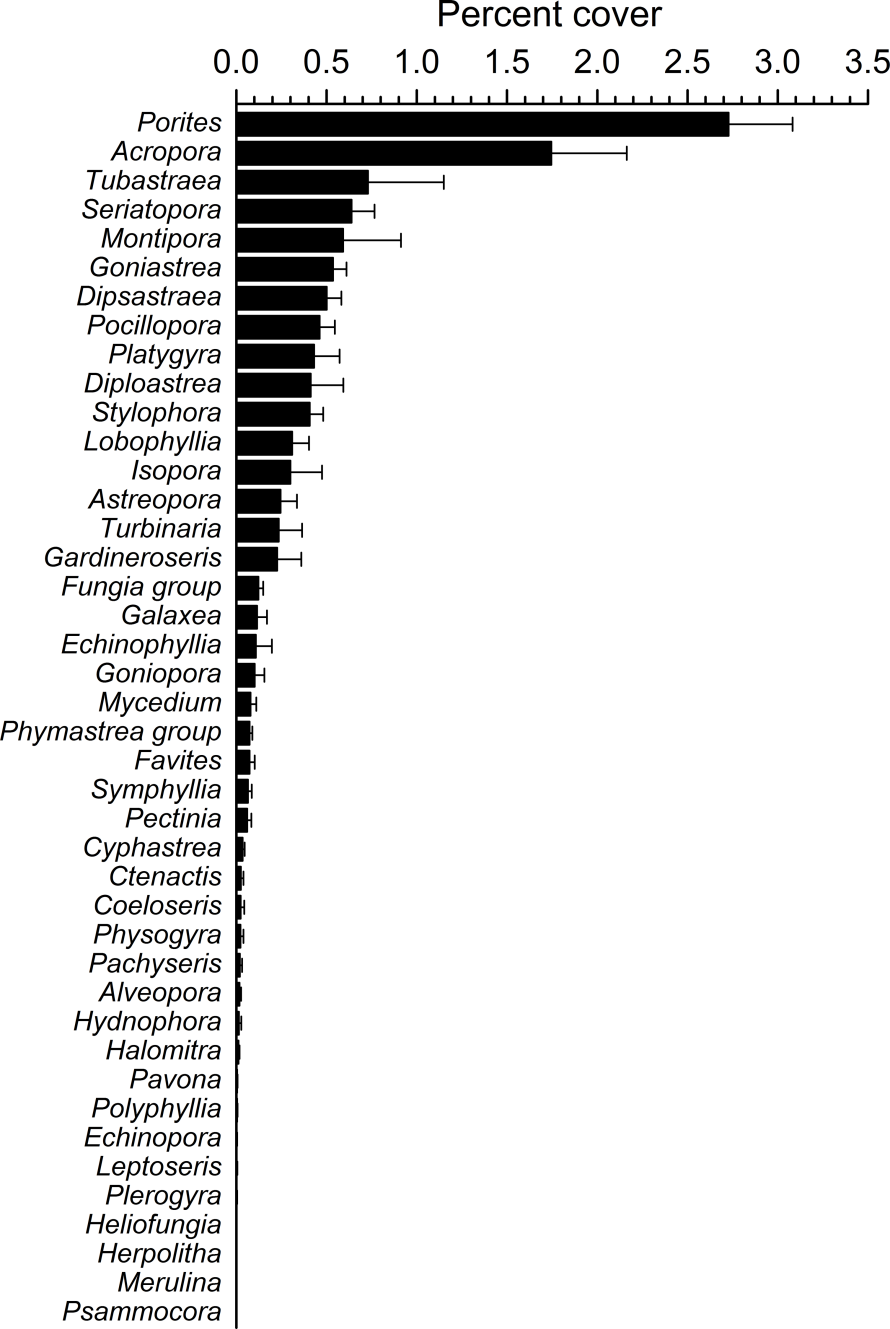
Overall scleractinian genera abundances. Mean percent cover (+SE) of each coral genus in the whole study area.

**Figure 5:**
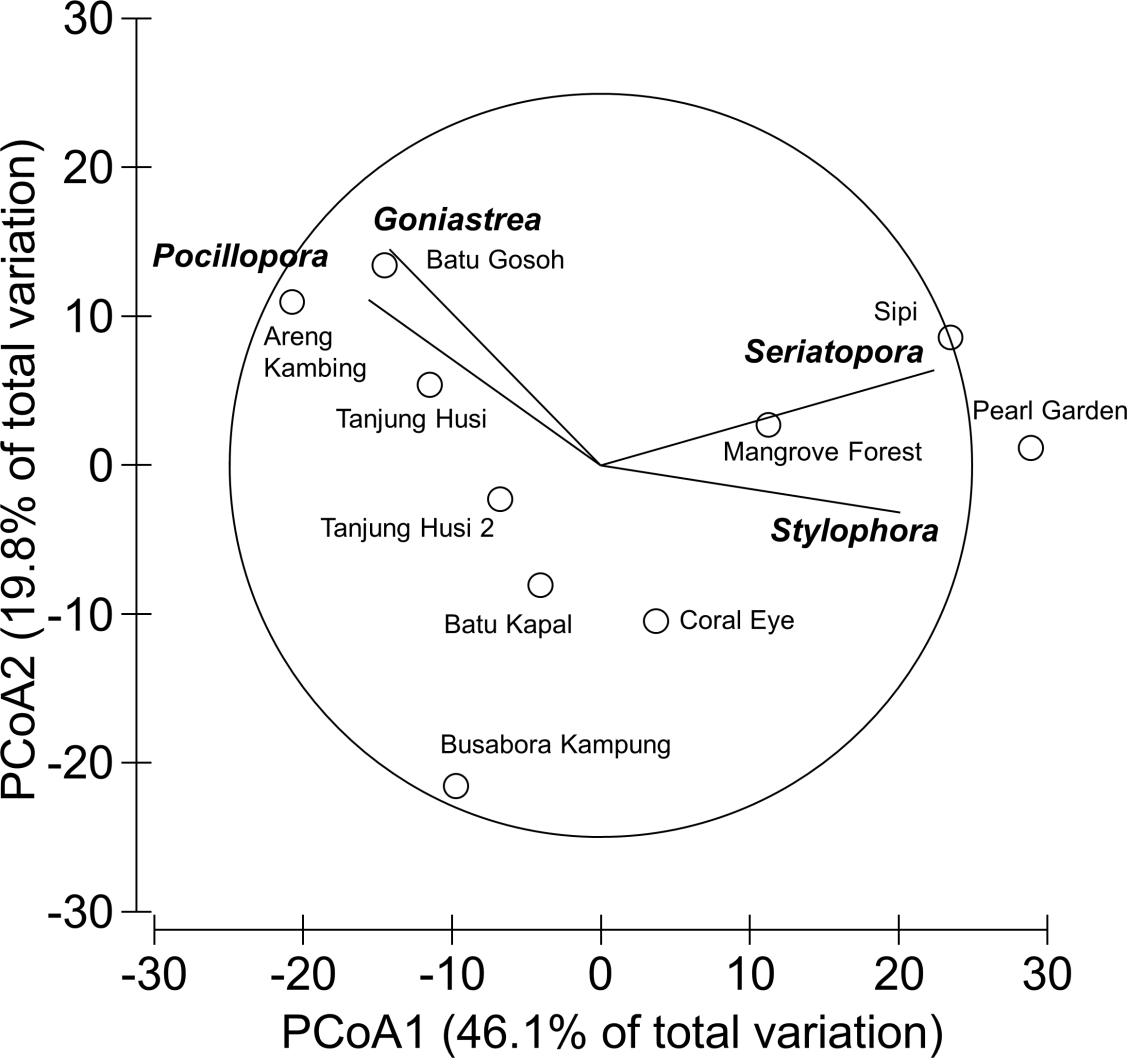
Similarity patterns of hard coral assemblages. The PCoA plot shows the multivariate similarity patterns of hard coral assemblages, analysed at the genus level, among study sites. Open circles represent the centroids of the similarities of assemblages found at each site. Superimposed vectors indicate the intensity and direction of the correlations of the genera selected by DistLM procedure (*P* < 0.05 in the marginal test).

Overall, 42 genera of hard corals were found. The most abundant genera were *Porites* (mean cover 2.73 ± 0.35%, up to 27.2%) and *Acropora* (mean cover 1.75 ± 0.42%, up to 42.0%; Fig. 4). Local high percent cover of *Tubastraea* (up to 54.4%) and *Montipora* (up to 43.8%) was also observed. Hard coral assemblages were very heterogeneous and variable among sites and depths (Site × Depth: *F*_18,118_ = 1.2787, *P* = 0.0044). Nevertheless, few hard coral genera significantly contributed to the observable differences among sites. Those that did, included: *Seriatopora*, *Stylophora*, *Goniastrea*, and *Pocillopora* (Fig. 5).

### Occurrence of coral diseases and other signs of compromised health

20 different types of coral disease and other compromised health statuses were recorded on 598 scleractinian colonies from 35 of the 42 genera identified at the different sites (Table 3). The scleractinian genera that hosted the higher number of diseases and other signs of compromised health were *Porites* followed by *Acropora*, with 14 and 11 categories recorded respectively (Table 3).

**Figure 6:**
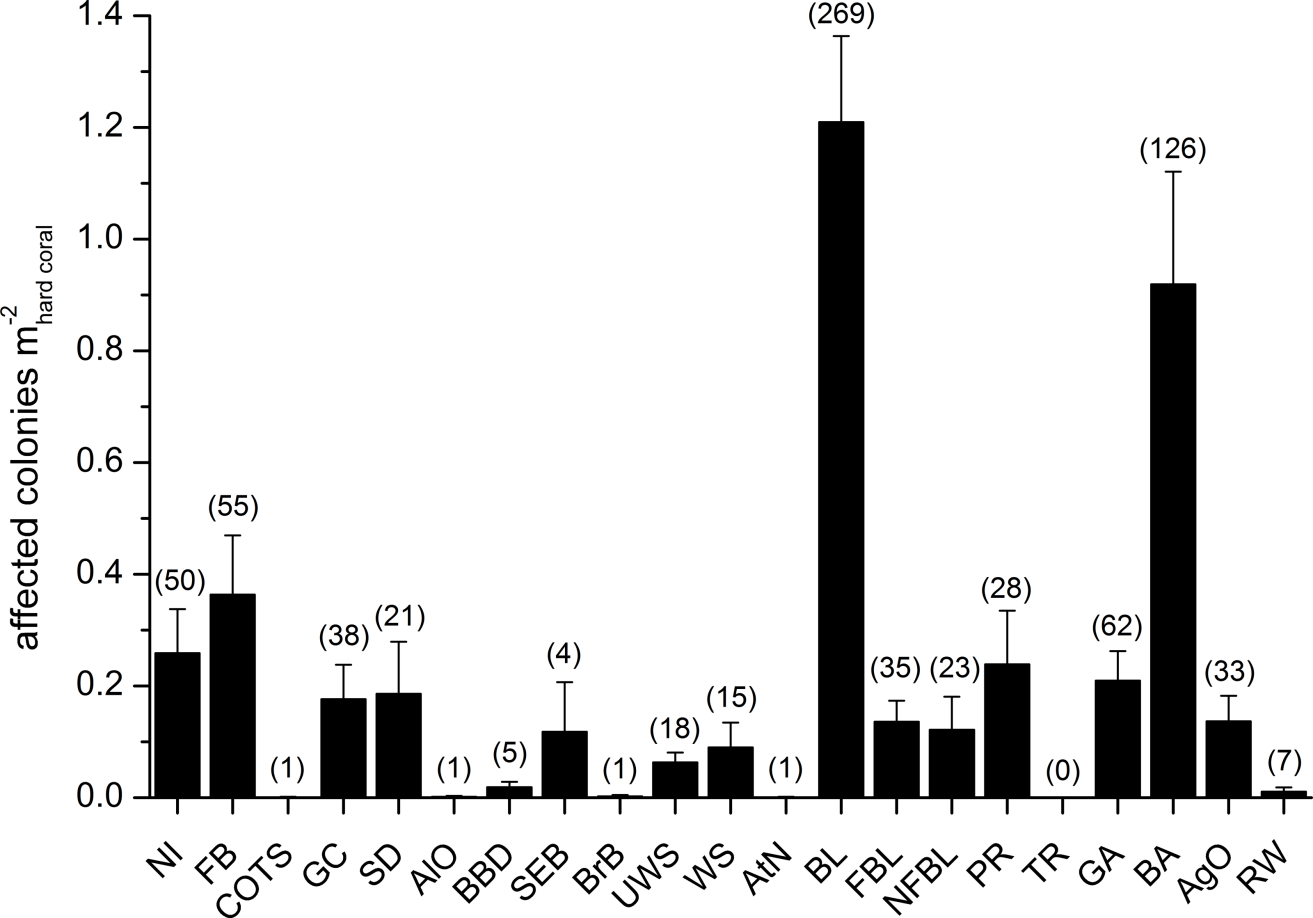
Overall occurrence of coral diseases and other signs of compromised health in the study area. Mean (+SE) number of affected colonies per seabed surface covered by hard corals for each category, disregarding the affected coral genus, in brackets the total number of occurrences (see Table 2 for the meaning of the acronyms).

**Figure 7:**
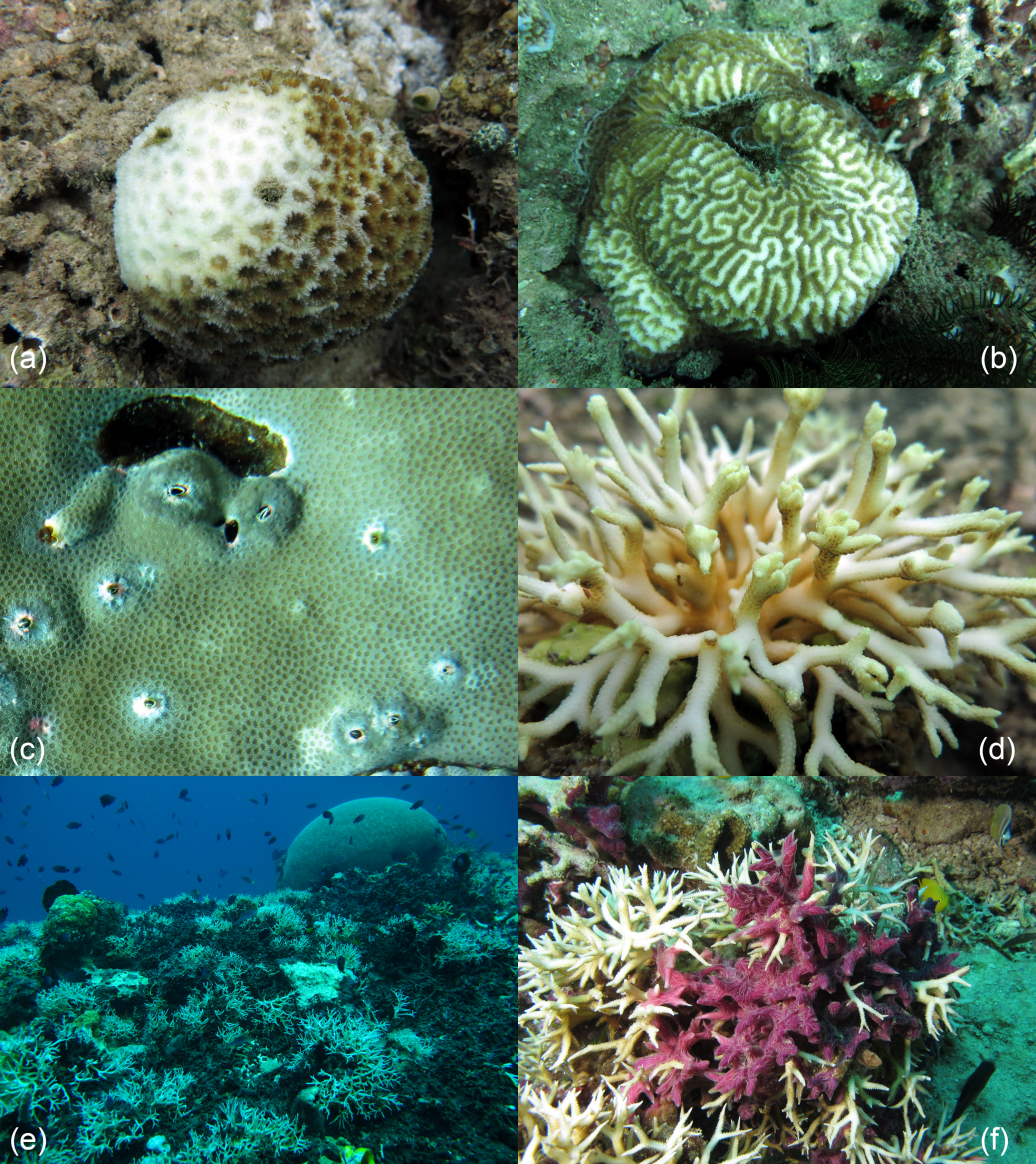
Examples of the most common health impairments found in the study area. Partially bleached colonies of *Dipsastraea* (A) and *Platygyra* (B); skeletal deformations caused by pyrgomatid barnacles in *Porites* (C); galls caused by cryptochirid crabs in *Seriatopora* (D); *Terpios hoshinota* infestation, mostly on *Seriatopora*, at Kahuko in 2011 (E); *Chalinula nematifera*, characterised by mauve coloration and white wavy filaments produced by symbiotic fungi, on *Seriatopora* (F).

Mean abundances and total number of occurrences of each category of coral diseases and other signs of compromised health are shown in Fig. 6. The most abundant type of compromised health recorded during this survey was coral bleaching (BL; Figs. 7A and 7B), followed by skeletal deformations caused by pyrgomatid barnacles (BA; Fig. 7C), damage caused by fish bites (FB), pigmentation response (PR) and galls caused by cryptochirid crabs (GA; Fig. 7D).

**Figure 8:**
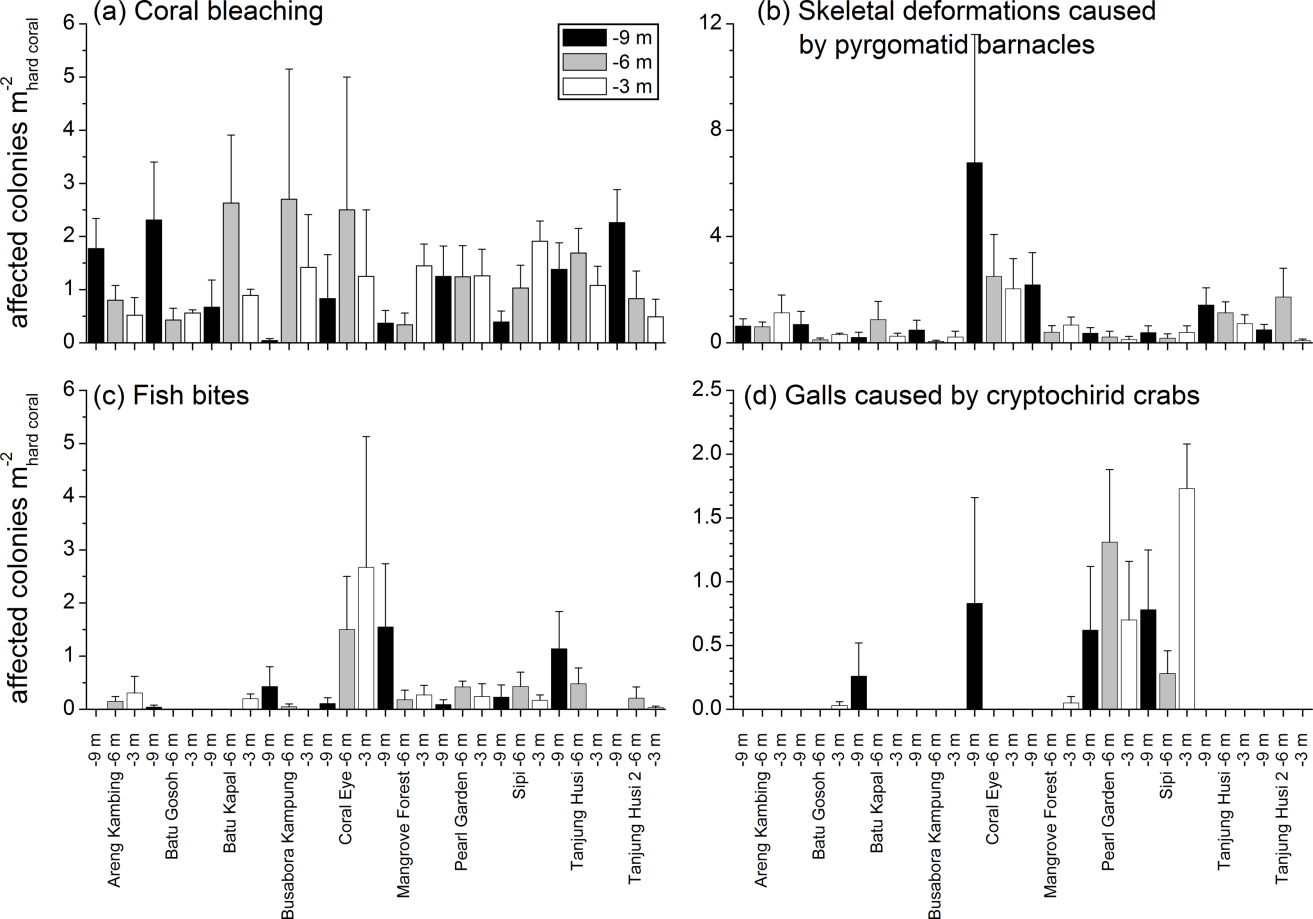
Spatial and depth distribution of coral bleaching (A), skeletal deformations caused by pyrgomatid barnacles (B), fish bites (C), and galls caused by cryptochirid crabs (D). Mean (+SE) number of affected colonies per seabed surface covered by hard corals at each site and investigated depth (−9, −6, −3 m).

No significant differences were recorded for bleaching across sites or depths, with instances recorded across 69% of the transects (mean 1.21 ± 0.15 affected colonies m${}_{\mathrm{hard~ corals}}^{-2}$) (Fig. 8A, Table 4). Bleaching was shown to affect 26 hard coral genera (Table 3), with the most affected genera being *Porites*, *Dipsastraea*, *Goniastrea*, *Platygyra*, *Seriatopora*, * Acropora* and *Pocillopora*.

Skeletal deformations caused by pyrgomatid barnacles were found in 51% of transects with a mean abundance of 0.92 ± 0.20 affected colonies m${}_{\mathrm{hard~ corals}}^{-2}$. Their distribution was heterogeneous, showing significant differences of abundance among sites (Fig. 8B, Table 4). Pyrgomatid barnacles were found to occur in 16 genera (Table 3); mostly colonising massive forms of corals belonging to the *Porites* genus.

**Table 3:** Diseases and affected corals. Diseases and other signs of compromised health affecting each scleractinian genus (see Table 2 for the meaning of the acronyms).

Coral genus	NI	FB	COTS	GC	SD	AlO	BBD	SEB	BrB	UWS	WS	AtN	BL	FBL	NFBL	PR	GA	BA	AgO	RW	Total
*Acropora*	+	+	+	+	+				+	+	+		+					+	+		11
*Alveopora*														+							1
*Astreopora*					+						+		+	+		+		+	+		7
*Coeloseris*	+												+	+				+			4
*Ctenactis*								+						+	+						3
*Cyphastrea*	+				+						+		+					+	+		6
*Diploastrea*															+				+		2
*Dipsastraea*					+					+			+	+	+			+			6
*Echinophyllia*															+						1
*Echinopora*													+								1
*Favites*	+									+			+		+			+			5
*Fungia* group					+								+	+		+			+		5
*Galaxea*	+							+					+								3
*Gardineroseris*	+					+												+	+	+	5
*Goniastrea*	+						+			+			+	+	+			+	+		8
*Goniopora*													+		+						2
*Halomitra*															+						1
*Hydnophora*													+					+			2
*Isopora*	+			+						+			+					+	+		6
*Lobophyllia*	+												+								2
*Montipora*	+									+	+	+	+								5
*Mycedium*	+														+						2
*Pachyseris*	+				+																2
*Pavona*					+					+			+	+				+			5
*Pectinia*													+		+				+		3
*Phymastrea* group					+								+					+			3
*Physogyra*	+																				1
*Platygyra*					+						+		+	+				+		+	6
*Plerogyra*	+												+								2
*Pocillopora*	+	+		+									+					+			5
*Polyphyllia*	+				+								+	+							4
*Porites*	+	+			+		+	+		+	+		+	+	+	+		+	+	+	14
*Seriatopora*	+												+				+		+		4
*Stylophora*	+			+									+				+		+		5
*Turbinaria*	+			+	+		+						+		+			+		+	8
Total	20	3	1	5	12	1	3	3	1	8	6	1	26	11	12	3	2	16	12	4	

Fish bites were found in 27% of transects with no significant difference of abundance among sites and depths (0.36 ± 0.11 affected colonies m${}_{\mathrm{hard~ corals}}^{-2}$; Fig. 8C, Table 4). Again, these were more typically associated with massive *Porites* colonies and to a lesser extent *Acropora* and *Pocillopora* (Table 3).

Signs of pigmentation response (PR) i.e., tissue discoloration, often bordering specific lesions or scars, were found in 16% of transects with a mean of 0.24 ± 0.10 affected colonies m${}_{\mathrm{hard~ corals}}^{-2}$. Their distribution was very heterogeneous, with significant differences found in abundance amongst both sites and depths (Table 4). However PR was only associated with three genera; *Acropora*, *Fungia* group and *Porites* (Table 3). It should be noted that PR (as classified in this study) can be caused by a number of factors such as specific coral borers, competitors, algal abrasion, fish bites, breakages, etc. and is therefore a type of “inflammatory” response suggesting a compromised health state but not itself a sign of disease (Beeden et al., 2008). Galls (GA), caused by cryptochirid crabs were encountered only at six of the sites surveyed (out of 10). GA occurred on 15% of transects, with a mean of 0.21 ± 0.05 affected colonies m${}_{\mathrm{hard~ corals}}^{-2}$. Occasionally differences of GA abundances occurred between depths (Fig. 8D, Table 4). GA were observed more commonly with branching *Seriatopora* and to a lesser extent *Stylophora* (Table 3).

A number of unidentified signs of tissue loss (named as NI in this manuscript), were found in 20 of the coral genera (Table 3) and at all sites (25% of transects, 0.26 ± 0.08 affected colonies m${}_{\mathrm{hard~ corals}}^{-2}$), randomly distributed between site and depth (Table 4).

Amongst less abundant categories of coral diseases and other signs of compromised health, cases of ulcerative white spot (UWS) were the most prevalent (found at nine out of 10 of the sites surveyed). UWS occurred on 11% of transects, with 0.06 ± 0.02 affected colonies m${}_{\mathrm{hard~ corals}}^{-2}$. Eight genera showed signs of UWS (Table 3), with *Porites* being the most commonly affected and significant differences between sites (Table 4).

**Table 4:** Effects of sites and depths on the distribution of coral diseases and other signs of compromised health. Summary of PERMANOVA tests on the abundance (affected colonies m${}_{\mathrm{hard~ corals}}^{-2}$) of the most common coral diseases and other signs of compromised health, analysed individually, according to the factors Site, Depth, and their interaction (Site × Depth). See Table 2 for the meaning of the acronyms.

	Site			Depth			Site × Depth			Res
	MS	*F*_9,118_	*P*	MS	*F*_2,18_	*P*	MS	*F*_18,118_	*P*	MS
NI	0.6817	0.7194	0.7332	0.3929	0.4257	0.6740	0.9229	0.9739	0.4935	0.9477
FB	2.7547	1.6906	0.0708	0.0266	0.0178	0.9836	1.4972	0.9189	0.5800	1.6294
GC	0.6276	1.1400	0.3154	0.5835	0.9926	0.4127	0.5879	1.0679	0.3522	0.5505
SD	1.0092	0.7667	0.7422	0.5151	0.3747	0.7379	1.3747	1.0443	0.3652	1.3164
UWS	28.8050	1.6294	0.0041^**^	17.7010	1.0192	0.4588	17.3660	0.9824	0.5456	17.6780
WS	0.2285	0.7742	0.7603	0.3539	1.1990	0.3630	0.2951	0.9999	0.4530	0.2952
BL	0.8230	0.2220	0.9942	1.6280	0.4293	0.6647	3.7922	1.0230	0.4229	3.7069
FBL	0.2643	1.2635	0.2339	0.0813	0.4067	0.6855	0.1997	0.9548	0.5131	0.2092
NFBL	0.4497	0.8634	0.6467	0.4337	0.8365	0.4997	0.5185	0.9955	0.4662	0.5208
PR	1.8391	1.5731	0.0843	4.0477	1.9376	0.1715	2.0911	1.7887	0.0144^*^	1.1691
GA	1.9667	6.7696	0.0003^***^	0.1365	0.2783	0.7676	0.4909	1.6896	0.0449^*^	0.2905
BA	16.6410	3.0347	0.0018^**^	8.0038	1.9467	0.1637	4.1084	0.7492	0.8109	5.4836
AgO	0.3706	1.2014	0.2655	0.6377	2.5486	0.0924	0.2501	0.8108	0.7901	0.3084

**Notes.**

* Indicated significant level of *P* < 0.05.

** Indicated significant level of *P* < 0.01.

*** Indicated significant level of *P* < 0.001.

Signs of gastropod corallivory (GC; e.g., due to *Drupella* spp.) were found in five coral genera and were more abundant on *Pocillopora*. Sediment damage (SD) affected 12 genera but with relatively low prevalence at each site (Table 3). Focal (FBL) and non-focal bleaching (NFBL, e.g., patches, stripes) together with aggressive overgrowth by sponges and other invertebrates (AgO), e.g., the coral-killing sponges *Terpios hoshinota* and *Chalinula nematifera*, were found on 11, 12 and 12 coral genera respectively (Table 3), again at relatively low prevalence (Fig. 6).

Interestingly, trematodiasis (TR) was not found on any of the reefs surveyed and black band disease (BBD) was only found on one colony of *Goniastrea*, two colonies of *Porites* and two of *Turbinaria.* Similarly, skeletal eroding band (SEB) was found only on a colony of *Ctenactis*, a colony of *Galaxea* and two colonies of *Porites*. Only one colony of *Acropora* and one of *Montipora* showed signs of brown band disease (BrB) and atramentous necrosis (AtN) respectively.

Other occasional findings included one *Acropora* affected by crown-of-thorns starfish (*Acanthaster planci*; COTS), one *Gardineroseris* showing signs of algal overgrowth (AlO), and a few colonies of *Gardineroseris*, *Platygyra*, *Porites* and *Turbinaria* infested by acoelomorph flatworms (RW), more precisely *Waminoa* sp. (Table 3; Fig. 6).

**Table 5:** Effects of sites and depths on the distribution of coral diseases assemblages. PERMANOVA test on the multivariate similarity patterns obtained by applying the zeroadjusted Bray-Curtis coefficient to square root transformed abundances of diseases and other signs of compromised health (affected colonies m${}_{\mathrm{hard~ corals}}^{-2}$) according to the factors Site, Depth, and their interaction (Site × Depth).

Source	df	SS	MS	F	*P*	Perms	Denominator
Site	9	48362	5374	2.3929	0.0001	9838	Res
Depth	2	3434	1717	0.6393	0.8225	9932	Site × Depth
Site × Depth	18	48361	2687	1.1964	0.0767	9783	Res
Res	118	264990	2246				
Total	147	365100					

### Spatial distribution patterns of diseases and other signs of compromised health

**Figure 9:**
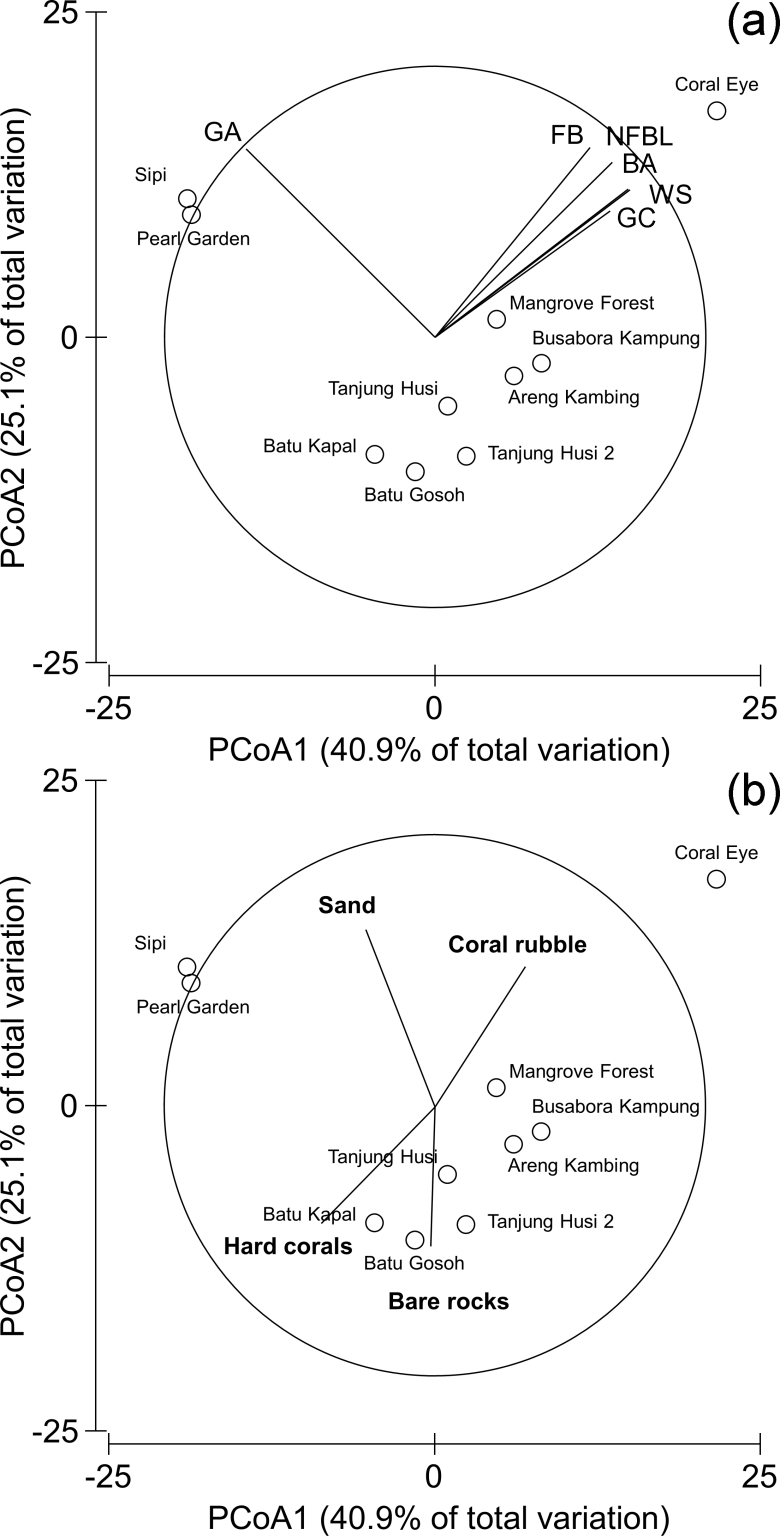
Similarity patterns of diseases and other signs of compromised health. The PCoA plot shows the multivariate similarity patterns of diseases and other signs of compromised health assemblages among study sites. Open circles represent the centroids of the similarities of assemblages found at each site. Correlation vectors superimposed on the PCoA plot represent: (A) diseases and compromised health categories significantly related with the similarity pattern (see Table 2 for the meaning of the acronyms); (B) substrate typologies significantly related with the similarity pattern (selected by DistLM procedure; *P* < 0.05 in the marginal test).

Patterns of similarities in diseases and other signs of compromised health revealed significant differences among sites, but not among depths (Table 5; Fig. 9A). The categories that significantly contribute to the observed similarity pattern were skeletal deformations caused by pyrgomatid barnacles, occurrence of corals showing signs of white syndromes, fish bites, non-focal bleaching and gastropod corallivory. These signs of compromised health increased in abundance closer to the Coral Eye site. In contrast, galls caused by cryptochirid crabs increased towards Sipi and Pearl Garden sites (Fig. 9A).

The observed spatial pattern were not correlated with the distribution of the benthic assemblages; however, there was a weak correlation with the abundance of sand, total hard coral, coral rubble (which increased towards Coral Eye) and the amount of bare rock (that characterised the volcanic cliff and rockslide at Batu Gosoh, Tanjung Husi and Tanjung Husi 2; Fig. 9B).

### Correlations between diseases and possible human and natural disturbances

According to information gathered from the local populace and previous surveys (Ponti et al., 2012), the most impacted sites in terms of mechanical disturbance, pollution and fishing, were Busabora Kampung (close to the homonymous village), Pearl Garden (located in the surroundings of a dismissed pearl farm), and Sipi (in front of the newly established metal mine) see Table 1. At Pearl Garden and Sipi, signs of recent blast fishing were evident. The most relevant recent storm happened a year earlier and hit the southeastern side of the island.

Few significant rank correlations between the abundances of diseases and other signs of compromised health were observed with regard to possible human and natural disturbances. Abundance of colonies affected by skeletal eroding bands increased with the intensity of bio-chemical disturbances (*ρ* = 0.6864, *P* = 0.0284) and decreased with distance from villages (*ρ* =  − 0.7647, *P* = 0.0100). Moreover, instances of damage caused by sedimentation together with observed instances of non-focal bleaching, decreased with regard to distance from the villages (respectively: *ρ* =  − 0.6933, *P* = 0.0262 and *ρ* = 0.7455, *P* = 0.0133), while skeletal deformations caused by pyrgomatid barnacles decreased with the intensity of bio-chemical disturbances (*ρ* =  − 0.6963, *P* = 0.0253).

## Discussion

The present study represents the first assessment of coral diseases and other signs of compromised health around Bangka Island at the centre of the Coral Triangle. Surveys were conducted at three depths (3, 6 and 9 m) at 10 random sites. Although the surveys were limited to shallow depths, this study provides a wide overview of coral health status in the investigated area and reveals new possible threats. Baseline surveys such as these are vital for management and mitigation of reef environments to allow for assessment of how different stressors are affecting reefs at different locations. Interestingly, results of this survey showed that the percentage of corals showing signs of ‘common’ occurring diseases such as black band disease, brown band disease, white syndrome and skeletal eroding band disease was relatively low compared to other studies (e.g., Willis, Page & Dinsdale, 2004; Dalton & Smith, 2006; Myers & Raymundo, 2009; Weil et al., 2012). Yet, the findings are more consistent with recent studies in other areas such as the Maldives (Montano et al., 2015; Montano et al., 2016) and south-east Sulawesi (Haapkylä et al., 2007; Haapkylä et al., 2009b). Infectious diseases, such as those mentioned above have been previously shown to be commonly promoted or exacerbated by mechanical injuries and predation activities (e.g., Winkler, Antonius & Renegar, 2004; Page & Willis, 2008; Nicolet et al., 2013). For example, brown band disease is generally associated with acroporids which is sustained by ciliates (Sweet & Bythell, 2012; Sweet & Séré, 2016) and promoted by mechanical scarring from coral predators such as *Acanthaster planci* (Katz et al., 2014) and *Drupella* spp. (Nicolet et al., 2013). The rarity of this disease around Bangka Island might be related to the low local abundance of such predators. Skeletal eroding band, also associated with ciliates but with a wider host range (Antonius & Lipscomb, 2001; Page & Willis, 2008; Sweet & Séré, 2016), was found to increase towards villages and their bio-chemical disturbances, supporting the hypothesis that its spread is favored by anthropogenic stresses that may compromise corals health (Page & Willis, 2008; Montano et al., 2016).

Coral bleaching was the most common sign of compromised health in the study area, affecting 26 genera. This fits current trends associated with reefs on a global scale (Berkelmans & Oliver, 1999; Carpenter et al., 2008; Burke et al., 2012; Sutthacheep et al., 2013). Although there appears to be no escape for corals from global increases in sea surface temperature (Baker, Glynn & Riegl, 2008), the susceptibility of corals to bleaching appears to vary considerably. For example differences have been reported between species (e.g., Hoeksema, 1991; Marshall & Baird, 2000; Loya et al., 2001; Montano et al., 2010; Hoeksema & Matthews, 2011) and locations (e.g., Pineda et al., 2013). The latter variation has been linked to differences in several regional environmental factors (e.g., light intensity and water flow rate) which appear to be influencing the outcome of bleaching intensity (Glynn, 1996; Brown, 1997). Indeed, it has long been ‘known’ that branching corals (e.g., acroporids and pocilloporids) are more sensitive to thermal stress than massive growth forms (Loya et al., 2001; Wooldridge, 2014). However, recent studies have shown quite the opposite can occur (Guest et al., 2012). Observing high levels of bleaching at any given time is often a worrying trend. However, the lack of major diseases in this region is a promising sign, as studies have shown that bleaching events on their own do not necessarily change the coral taxonomic community structure despite often resulting in a reduced amount of total coral cover (Guest et al., 2016).

Next to bleaching, the second most common sign of compromised health around Bangka Island was skeletal deformations caused by pyrgomatid barnacles. Corals with massive growth forms (*Porites* and *Platygyra* for example), showed the highest abundances of these skeletal deformations. Interestingly, although such organisms are present on reefs throughout the world, few studies focus on assessing the potential damage they can do to individual colonies (Frank et al., 1995). These coral-inhabiting barnacles are unable to bore directly into the host, but to varying extent, they are able to inhibit or regulate their host’s skeletal growth (Anderson, 1992). Few species are considered able to adversely affect their hosts, nevertheless infestations may damage especially finely branched scleractinian corals (Ross & Newman, 1995). Although this study does not assess the effects high abundances of these organisms have on these reefs, the high abundances found warrants further study to assess if these should be recorded in future baseline surveys of reef health.

Similar to above, the abundances of fish bites, including parrotfish, butterflyfish, filefish, pufferfish, triggerfish, and damselfish families, for example are rarely characterised and therefore measured in coral health surveys. This is despite several studies suggesting that corallivorous fishes may be vectors for coral disease (Rogers, 2008). Indeed numerous fish species have been documented to target feeding on coral lesions when present and this has facilitated the hypotheses that they can spread the infectious agents from colony to colony (Chong-Seng et al., 2011). Here, the majority of fish bites observed were associated with massive *Porites* with only few examples associated with colonies of *Acropora* and *Pocillopora*. Here instead of highlighting the potential of disease occurrence due to fish bites, we argue that an intermediate level of predation by fishes on hard coral should actually be considered an index of a balanced fish assemblages in almost all healthy coral reef environments (Cole, Pratchett & Jones, 2008).

In contrast, possible outbreaks of the corallivorous gastropods *Drupella* spp. are raising concerns not only for their direct predatory effects on their preys, but also for their feared involvement as vector in some infectious diseases (e.g., Turner, 1994; Antonius & Riegl, 1998; Onton et al., 2011; Nicolet et al., 2013). Luckily, cases of *Drupella* around the island are still relatively few yet we recommend they should still be monitored over time.

A commonly overlooked group of associated fauna affecting the morphology of corals consists of cryptochirid crabs that live in corals of *Seriatopora* and related genera. These tiny crabs are obligate symbionts of living scleractinian corals and have been found to feed on coral tissue and mucus along with inducing gall formation (Kropp, 1990; Terrana et al., 2016). Here, high abundances of cryptochirid crabs were observed at Pearl Garden (the site of an old pearl farm) and Sipi (the location of an active metal mine) (Fig. 9A). Moreover, both sites were also characterised by recent signs of blast fishing. Although the rank correlation between galls caused by cryptochirid crabs and human disturbances was not significant, the presence of many cases in the study area, especially in the most disturbed sites, deserves further investigations. Gall crabs can perhaps also be found in other coral species in the research area, since in nearby Lembeh Strait (North Sulawesi), cryptochirids were observed to occur abundantly in a single, large coral colony of *Pavona clavus* (Hoeksema & Van der Meij, 2012).

Although coral injuries due to acoelomorph flatworms have not yet been reported, these species have been hypothesised to cause a shading effect which may result in a negative impact on the corals photophysiology (Haapkylä et al., 2009a) in addition to displacement of the corals surface mucus layer (Naumann et al., 2010). In this instance, only *Waminoa* sp. were observed, supporting a recent study which showed the presence of these species infesting mushroom corals in the same region, the Lembeh strait (Hoeksema & Farenzena, 2012). The effect these flatworms have on corals specifically when high infestations occur remains unknown. Finally, some instances of coral-killing sponges such as *Terpios hoshinota* and *Chalinula nematifera* were recorded overgrowing a variety of otherwise healthy corals within the surveyed transects. In Indonesia, the spread of the cyanobacteriosponge *T. hoshinota* in particular is worrying as this sponge has been linked to outbreaks of the so-called ‘black disease’ first observed by De Voogd, Cleary & Dekker (2013). Such infestations by *T. hoshinota* may have severe and persistent effects on reef areas within this region and on a global scale, however to date outbreaks appear to be spatially well defined and limited (Madduppa et al., 2015; Elliott et al., 2016; Van der Ent, Hoeksema & De Voogd, 2016). During our first visit of these reefs, back in September 2011, a wide area of reef off the Kahuku village (6 m depth) was observed to be affected by *T. hoshinota* (M Ponti, pers. obs., 2011; Fig. 7E). Confirmation of this discovery (by DNA sequencing of the sponge) should be undertaken in order to track the westward widening of this species geographical range (Madduppa et al., 2015). In addition, at the same site and occasion several other corals were observed to be affected by *C. nematifera* (Fig. 7F). *C. nematifera* is similarly reportedly to be spreading fast throughout the Indo-Pacific (Ávila & Carballo, 2009) and is thought to have been introduced to this region, possibly by fouling from ships. The presence of *C. nematifera* in the study area and along eastern Sulawesi was recently documented by Rossi et al. (2015), nevertheless quantitative studies are urgently needed to assess the severity of these sponge outbreaks.

Interestingly, although most studies of this kind focus on the coral genera which show presence of disease signs or other compromised health, few highlight those genera which appear disease-free. In this instance, the majority of genera which showed no signs of compromised health were also those with very low abundances such as *Heliofungia*, *Herpolitha*, *Leptoseris*, *Merulina*, *Psammocora* and *Symphyllia.* However such findings may simply be down to the low occurrence of these genera. In contrast, *Tubastraea* which was one of the most abundant genera of corals found at our sites was also shown to have a lack of apparent instances of compromised health and further studies should assess if this trend is seen throughout the corals range.

To conclude, although the study area is relatively undisturbed as far as tourism development, impact on the area by other human activities appear to be having a significant effect on coral health and this warrants further longer term study. Of particular concern is the increase in mining which is occurring in this area along with other off shore islands throughout Sulawesi (Edinger, Siregar & Blackwood, 2007; Caras & Pasternak, 2009; Lasut et al., 2010). However, directly linking such activities to coral health status can be challenging (Rogers, 1990; Bruckner & Bruckner, 1997; Fabricius, 2005; Voss & Richardson, 2006a; Haapkylä et al., 2011; Erftemeijer et al., 2012; Pollock et al., 2014; Heintz, Haapkylae & Gilbert, 2015).

Regardless of asserting the cause of reef decline, the provision of baseline surveys for monitoring coral health status lay the foundations to assess the effects of any such anthropogenic and/or natural effects on reefs over future years. Therefore, such activities should continue to be undertaken with the understanding that return visits are scheduled and conducted in the same manner to allow for direct comparisons between data sets.

##  Supplemental Information

10.7717/peerj.2614/supp-1 Data S1 Raw data For each sampling transect (10 × 2 m), within all sites and depths (−3, −6 and −9 m), coral diseases and other signs of compromised health, in terms of number of affected colonies per square metre of hard corals (see Table 2 for the meaning of the acronyms), substrates (coral rubble, sand and rocks), main benthic groups and hard coral genera (as of percent cover) have been reported.

## Additional Information and Declarations

### Field Study Permissions

The following information was supplied relating to field study approvals (i.e., approving body and any reference numbers): A research permit was granted prior to undertaking the survey work by the authority of Lihunu, Likupang eastern districts, Minahasa northern district government (Permit ID: 211/2019/SPP/IX-2013 issued at Lihunu on the 28th September 2013).

### Data Availability

The following information was supplied regarding data availability: The raw data has been supplied as a Data S1.
